# Study of the Antioxidant Effects of Coffee Phenolic Metabolites on C6 Glioma Cells Exposed to Diesel Exhaust Particles

**DOI:** 10.3390/antiox10081169

**Published:** 2021-07-23

**Authors:** Laura Botto, Alessandra Bulbarelli, Elena Lonati, Emanuela Cazzaniga, Michele Tassotti, Pedro Mena, Daniele Del Rio, Paola Palestini

**Affiliations:** 1 School of Medicine and Surgery, University of Milano-Bicocca, 20900 Monza, Italy; laura.botto@unimib.it (L.B.); alessandra.bulbarelli@unimib.it (A.B.); elena.lonati1@unimib.it (E.L.); emanuela.cazzaniga@unimib.it (E.C.); 2 POLARIS Centre, University of Milano-Bicocca, 20126 Milano, Italy; 3 Bicocca Center of Science and Technology for Food, University of Milano-Bicocca, Piazza della Scienza, 2, 20126 Milano, Italy; 4 Human Nutrition Unit, Department of Food and Drug, University of Parma, 43121 Parma, Italy; michele.tassotti@studenti.unipr.it (M.T.); pedromiguel.menaparreno@unipr.it (P.M.); daniele.delrio@unipr.it (D.D.R.); 5 School of Advanced Studies on Food and Nutrition, University of Parma, 43121 Parma, Italy

**Keywords:** air pollution, phytochemical, coffee phenolic metabolites, oxidative stress, ROS, neurodegenerative diseases

## Abstract

The contributing role of environmental factors to the development of neurodegenerative diseases has become increasingly evident. Here, we report that exposure of C6 glioma cells to diesel exhaust particles (DEPs), a major constituent of urban air pollution, causes intracellular reactive oxygen species (ROS) production. In this scenario, we suggest employing the possible protective role that coffee phenolic metabolites may have. Coffee is a commonly consumed hot beverage and a major contributor to the dietary intake of (poly) phenols. Taking into account physiological concentrations, we analysed the effects of two different coffee phenolic metabolites mixes consisting of compounds derived from bacterial metabolization reactions or phase II conjugations, as well as caffeic acid. The results showed that these mixes were able to counteract DEP-induced oxidative stress. The cellular components mediating the downregulation of ROS included extracellular signal-regulated kinase 1/2 (ERK1/2), nuclear factor erythroid 2-related factor 2 (Nrf2), heme oxygenase-1 (HO-1), and uncoupling protein 2 (UCP2). Contrary to coffee phenolic metabolites, the treatment with N-acetylcysteine (NAC), a known antioxidant, was found to be ineffective in preventing the DEP exposure oxidant effect. These results revealed that coffee phenolic metabolites could be promising candidates to protect against some adverse health effects of daily exposure to air pollution.

## 1. Introduction

Air pollution is a prevalent source of environmentally induced inflammation and oxidative stress. Each year millions of people are exposed to levels of air pollution above promulgated safety standards [1]. Diesel exhaust particles (DEPs) are a major constituent of near-road and urban air pollution and are commonly used as a surrogate model of air pollution in health effects studies [2,3]. Much evidence suggests that exposure to air pollution can increase the risk of fatal stroke, cause cerebrovascular damage, and induce neuroinflammation and oxidative stress that may trigger neurodegenerative diseases [4,5,6,7,8]. Once inhaled, DEPs can enter the circulation and translocate to tissues throughout the body, reaching also the brain by crossing the blood–brain barrier (BBB) [9]. Indeed, exposure to DEPs was shown to alter BBB function through oxidative stress [10]. Oxidative stress develops when there is an imbalance between the production of reactive oxygen species (ROS) and the availability of antioxidant defences [11].

DEPs consist of a carbon core and heavy hydrocarbons derived from fuel and lubricant oils and hydrated sulfuric acid derived from the fuel sulfur. In addition, DEPs have polycyclic aromatic hydrocarbons (PAHs) adsorbed to them [12]. This is supported by the observations reported in a growing number of studies that DEPs may exert their toxicity by inducing oxidative stress [13,14].

Even though the mechanisms responsible for the production of ROS following DEP exposure are still poorly understood, we were able to show that the MEK-ERK1/2 pathway and Nrf2, concomitantly with a significant increase in HO-1 levels, are involved in regulating the antioxidant strategies to compensate the oxidative status induced by DEP treatment [15]. Literature data suggested that ERK1/2 activation could trigger Nrf2 phosphorylation, facilitating its translocation into the nucleus and the consequential increased synthesis of a number of phase II proteins [16,17,18,19]. Activated Nrf2 can act as a master regulator of several genes for antioxidant enzymes and detoxifying enzymes, by binding activated antioxidant response elements. Nrf2 appears to be a key regulator of the cellular response to oxidative stress and HO-1 is one of Nrf2 target genes [20,21]. Among the redox-sensitive inducible enzymes, HO-1 serves as a protective gene due to its antioxidant properties. Indeed, HO-1 is an enzyme highly upregulated under oxidative stress conditions, and it represents the rate-limiting step in heme degradation that produces antioxidant molecules [19]. Furthermore, it is known that ultrafine particulate pollutants localize in mitochondria, where they induce major damage, and this may contribute to oxidative stress [22].

There are several studies indicating that respiratory uncoupling proteins (UCPs) can uncouple ROS production. UCP2 and UCP3 can attenuate the mitochondrial ROS production and thus protect cells against oxidative damage [23,24]. Andrews and Horvath [25] demonstrated that increased expression of UCP2 could reduce the ROS production and oxidative stress in the tissues of mice and can consequently extend the lifespan of the animals. These observations agree with the hypothesis of “mitochondrial uncoupling to survive”. In particular, over-expression of UCP2 was reported to be neuroprotective against oxidative stress in vivo and in vitro [26].

There is considerable current interest in the modulation of ROS by phytochemicals, and in particular, the most important intermediates by which (poly) phenols mediate the downregulation of ROS include HO-1 and UCPs [27]. Indeed, recent studies have highlighted the key neuroprotective actions of (poly) phenols found in fruit, vegetables, and plant-derived beverages [28,29,30]. Hydroxycinnamic acids are phenolic acids widely found in coffee and other plant-based products such as potatoes, apple, artichoke, wine, and cereals. Among hydroxycinnamates, ferulic and caffeic acids are the most abundant in plant-based foods and are often bound to quinic acid to form feruloylquinic or caffeoylquinic acids, also known as chlorogenic acids (CGAs) [31,32]. Coffee is the main dietary source of CGAs in many countries [33,34], as it is one of the most widely consumed hot beverages in the world. A single serving provides between 20 and 675 mg of CGAs, depending on the variety, roasting, extraction procedure, and volume consumed [35,36,37] and regular consumers can easily have an intake in excess of 1 g per day [38,39]. Upon consumption, the bulk of coffee CGAs reaches the colon, where they are subjected to transformation into other phenolic acids by gut microbiota. Then, these phenolic catabolites undergo methylation, sulfation, and glucuronidation once absorbed, at both the intestinal and hepatic level (phase II metabolism) [31,32,40,41,42,43]. These conjugated phenolic metabolites derived from coffee consumption may be able to cross the BBB and exert neuroprotective effects [44]. During this passage, the conjugated form may be metabolized back to the parent aglycone, which then enters the central nervous system [45]. Nevertheless, the antioxidant role of main circulating coffee phenolic metabolites in neural cells have not been tested to date.

On the basis of these assumptions, we assess the oxidant effects of DEPs through in vitro experiments using C6 glioma cells, evaluating the possible protective role of coffee phenolic metabolites. Based on the physiological concentrations for the main circulating metabolites, two different mixes consisting of coffee phenolic metabolites derived from gut microbiota reactions (by specific enzymes belonging to the microbiome) and phase II conjugations (by enzymes engaged in the detoxification from xenobiotics) are tested. In addition, the caffeic acid antioxidant property is independently assessed since it is not conjugated by phase II enzymes, and therefore it is more likely to enter the central nervous system [45,46]. We carry out an analysis to explore the antioxidants’ effects of coffee metabolites under oxidative stress induced by DEPs, evaluating proteins involved in mediating the ROS downregulation, such as ERK1/2, Nrf2, HO-1 and UCP2.

## 2. Materials and Methods

### 2.1. Materials

All commercial chemicals were of the highest available grade and were purchased from Sigma Chemical Co. (Milano, Italy). All the stock solutions for cell culture were from Euroclone (Celbio, Milano, Italy). Precision Plus Protein Standards (All Blue) were from Bio-Rad (Milano, Italy). The complete protease inhibitor cocktail was from Roche Diagnostics S.p.A (Milano, Italy). Primary antibodies of anti-ERK1/2 were from Cell Signaling Technology (Danvers, MA, USA); anti-Nrf2 was from R&D Systems (Minneapolis, MN, USA); anti-HO-1 was from Santa Cruz Biotechnology Inc. (Santa Cruz, CA, USA); anti-UCP2 was from Byorbyt (Cambridge, UK); anti-beta Actin loading control, anti-mouse or anti-rabbit (HRP)-conjugated secondary antibodies, and ECL SuperSignal detection kit were from Thermo Fisher Scientific (Milano, Italy). Dihydrocaffeic acid, dihydroferulic acid, dihydroferulic acid-4′-sulfate, ferulic acid-4′-sulfate, caffeic acid-3′-glucuronide, caffeic acid-4′-glucuronide, and dihydrocaffeic acid-3′-glucuronide were purchased from Toronto Research Chemicals (Toronto, ON, Canada), while caffeic acid was from Sigma-Aldrich (St. Louis, MO, USA).

### 2.2. Cell Culture

Rat C6 glioma cells were purchased from the American Type Centre Collection (ATCC, Manassas, VA, USA). Cells were seeded in 96 multiwell plates, with a density around 5000 cells per well, and maintained at 37 °C, 5% CO_2_ in Dulbecco’s modified Eagle’s medium containing 10% fetal bovine serum, 1% penicillin and streptomycin, and 1% L-glutamine. After 24 h, cells were treated at 80% confluence [47].

### 2.3. Determination of Intracellular Reactive Oxygen Species (ROS)

Intracellular ROS production was estimated by using 2,7-dichlorofluorescein diacetate (DCFDA) as a probe [48]. DCF-DA diffuses through the cell membrane, where it is enzymatically deacetylated by intracellular esterases to the more hydrophilic nonfluorescent reduced dye dichlorofluorescin. In the presence of reactive oxygen metabolites, nonfluorescent DCFH rapidly oxidized to highly fluorescent product DCF. Based on the method setup, after performing the experiments (as described below), C6 glioma cells were incubated with 10 μM DCFH-DA in serum-free medium for 1 h at 37 °C. The formation of DCF was measured at the excitation wavelength of 485 nm and an emission wavelength of 535 nm by using a fluorescence spectrometer (Tecan Infinite^®^ M200 Pro). ROS production was normalized as a percentage of control.

### 2.4. Determination of Cells Viability

Viability of C6 glioma cells was determined by MTT (3-[4,5-dimethylthiazol-2-yl]-2,5-diphenyl-2H-tetrazolium bromide) assay [48]. Based on the method setup, after the different treatments (as described below), MTT stock solution (5 mg/mL) was added to each well to a final concentration of 1.2 mM, and cells were incubated for 3 h at 37 °C. The functional mitochondrial succinate dehydrogenases in survival cells can convert MTT to formazan that generates a blue colour. The accumulation of formazan directly reflects the activity of mitochondria as an indirect measurement of cell viability. Lastly, MTT solution was removed, and the reaction was stopped by adding EtOH. After 30 min under stirring, the optical density was measured at 570 nm with 630 nm as a reference, and cell viability was normalized as a percentage of control.

### 2.5. Method Setup with Tert-Butylhydroperoxide (tBHP) and N-Acetylcysteine (NAC)

tBHP is a well-known cytotoxin and oxidative agent that induces oxidative stress [49]. In fact, tBHP is a substrate of glutathione peroxidase known to interfere with the glutathione-dependent antioxidant defences of the cell. tBHP exposure determines a de-crease in reduced glutathione (GSH) levels together with an increase in oxidized glutathione (GSSG) level, potentially cytotoxic [50]. It is known that a dose of 50 µM of tBHP is effective in evoking a significant increase in ROS generation already after 20 min, in a human hepatoma cell line (HepG2) [51]. Therefore, four different sets of C6 glioma cells were incubated with four different doses of tBHP (50, 100, 200, 500 µM) for 30 min to determine the maximum production of ROS caused by tBHP treatment. The resulting cells were rinsed with PBS, and intracellular ROS production was estimated by using DCFDA as a probe. In experiments carried out in parallel, the viability of C6 glioma cells was determined by MTT assay.

Based on the results, the method used was considered reliable for the evaluation of oxidative stress in our cellular model. Thus, we were able to test the antioxidant effect of NAC, a well-known antioxidant molecule. In C6 glioma cells, pretreatment with 5 mM NAC for 1 h significantly reduced events associated with oxidative stress-induced cell death [52]. Therefore, two different sets of C6 glioma cells were exposed to 5 mM NAC for a short and a long time duration (2 and 24 h, respectively) before tBHP treatment. The assessments of intracellular ROS production by the DCFDA staining method and of cell viability by MTT assay were carried out to determine the optimal dose and time of NAC treatment, which can counter tBHP-induced ROS production. Based on the results, the method used was considered reliable for assessing the antioxidant effect in our cellular model.

### 2.6. Assessment of Diesel Exhaust Particles (DEP) Effects

Cells were treated with DEP SRM1650b (Standard Reference Material, National Institute of Standards and Technology, Gaithersburg, MD USA), a diesel particulate with mean aerodynamic diameter of 0.18 mm reach in PAHs (National Institute of Standard and Technologies; www.nist.gov/srmors/certificates/1650b.pdf?CFID=7862989&CF-TOKEN=2c41aaa9d4941743-95BE052A-B7AF-FEC3-6800359C67A97E13, accessed on 27 September 2006), which is used in literature as a model of ultrafine particulate matter [53]. Diesel particles were suspended at different concentrations (25 or 50 µg/mL) in culture medium supplemented with 0.00005% Tween-20 to allow for proper particles suspension. Based on in vivo models of near road and occupational exposure (0.5 and 2 mg/m^3^), Levesque and colleagues [53] calculated that an in vitro concentration of about 5–50 µg/mL of nanometre-sized particles falls within the current estimates of what might reach the brain. Although the precise amount of particulate matter (PM) reaching the brain is currently unknown, studies have demonstrated that 0.01–0.001% of inhaled nanometre-sized iridium and carbon particulate remain in the brain 24 h after exposure [54]. A dose of 25 µg/mL was chosen, as it is an intermediate dose, and it is the dose responsible for major significant changes in the in vitro experiments using C6 glioma cells [15]. Immediately before treatment, DEP suspensions were sonicated for 5 min by means of Bransonic1 (Ultrasonic Cleaner Branson 2510) to obtain a proper particle dispersion; a dynamic light scattering (Brookhaven Instruments Corporation, Holtsville, NY USA) analysis confirmed the particles’ dimensions as measured by the manufacturer. Briefly, eight different sets of C6 glioma cells were exposed to 25 μg/mL DEPs for different times (1 h, 3 h, 6 h, 8 h, 10 h, 12 h, 14 h, 17 h) to determine the maximum production of ROS caused by DEP treatment. The resulting cells were rinsed with PBS, and intracellular ROS production was estimated by using DCFDA as a probe. In experiments carried out in parallel, viability of C6 glioma cells was determined by MTT assay. Based on the results, we designed the in vitro experiments, and C6 glioma cells were incubated with 25 µM DEPs for 12 h.

### 2.7. Coffee Phenolic Metabolites

**Figure 1:**
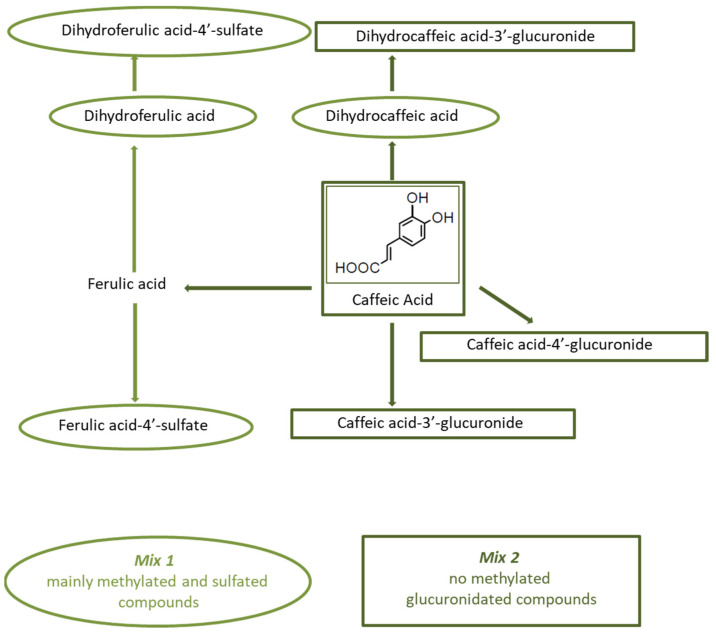
Schematic representation of the different coffee mixes.

Two solutions (i.e., mixes) consisting of coffee phenolic metabolites were prepared. Mix 1 contained dihydrocaffeic acid, dihydroferulic acid, dihydroferulic acid-4′-sulfate and ferulic acid-4′-sulfate (mainly methylated and sulfated compounds), while Mix 2 contained caffeic acid, caffeic acid-3′-glucuronide, caffeic acid-4′-glucuronide, and dihydrocaffeic acid-3′-glucuronide (no methylated, glucuronidated compounds) (Figure 1). The metabolites were tested at physiological concentrations (0.5–2 μM), as previously reported [40,55]. These substances had to be dissolved in DMSO before being diluted in DMEM medium, and the maximum dose that was compatible with the effects of DMSO was used. In order to check the effects of DMSO, two different sets of C6 glioma cells were incubated with two different doses of DMSO, i.e., 0.85% and 1.7% in DMEM. The dose of 0.85% DMSO in DMEM corresponded to the concentration of DMSO contained in 0.5 µM Mix 1 and in 1 µM Mix 2, while the dose of 1.7% DMSO in DMEM corresponds to the concentration of DMSO contained in 1 µM Mix 1 and in 2 µM Mix 2. Cells were incubated for 48 h at 37 °C, and viability was determined by MTT assay. The dose of 0.85% DMSO was found to be nontoxic to the cells. Accordingly, two different sets of C6 glioma cells were incubated with 0.5 µM of each compound present in Mix 1 or 1 µM of each compound in Mix 2 for 48 h at 37 °C before pro-oxidant treatments. The antioxidant activity of 1 μM caffeic acid was also assessed. Assessment of intracellular ROS production by DCFDA staining method and of cell viability by MTT were carried out both for the mixes and for caffeic acid.

### 2.8. SDS-PAGE Electrophoresis and Immunoblotting

Following DCFDA assessment, cells in each well were lysed with a denaturizing buffer (2% SDS lysis, 50 mM Tris-HCl, pH 6.8 plus protease inhibitor cocktail and phosphatase inhibitors) and stored at −20 °C until immunoblotting analysis. After total protein amount evaluation by means of Bicinchoninic acid assay, 25 µg of proteins was loaded on SDS-PAGE (10% polyacrylamide gel) and submitted to electrophoresis. Subsequently, proteins were transferred to nitrocellulose membranes and were revealed by Ponceau staining to assess proper transfer. Blots were washed with TBS and blocked for 1 h in TBS-T/5% milk or 3% BSA. After blocking, blots were incubated overnight with the primary antibody diluted in a blocking solution (anti-phospho ERK1/2 1:10,000, anti-ERK1/2 1:5000, anti-Nrf2 1:500, anti-HO-1 1:200, anti-UCP2 1:500, anti-β-actin 1:1000), and then for 1.5 h with HRP-conjugated anti-rabbit or anti-mouse IgG. Proteins were detected by ECL with the SuperSignal detection kit and analysed with ImageQuant™ 800 (GE Healthcare Life Sciences, Piscataway, NJ USA), with the program 1D gel analysis. The band intensity of the different proteins was normalized to the band intensity of the corresponding actin, and each protein was normalized as a percentage of control.

### 2.9. Statistical Analysis

Biochemical determinations were obtained from at least three independent experiments. All the values were expressed as mean ± SE. Statistical differences were tested by one-way ANOVA and t-test. A difference was considered significant at the 95% level (*p* < 0.05).

## 3. Results

### 3.1. Method Setup with tBHP and NAC

We used C6 glioma cells as a cellular model. Indeed, the C6 glioma cell line exhibits properties of both astrocytes [56] and oligodendrocytes [57], which are widely used in neurobiological research. Moreover, this cell line has already been used to test the oxidant toxicity of several components [58], because it exhibits results of being sensitive to oxidative stress [15,59].

**Figure 2:**
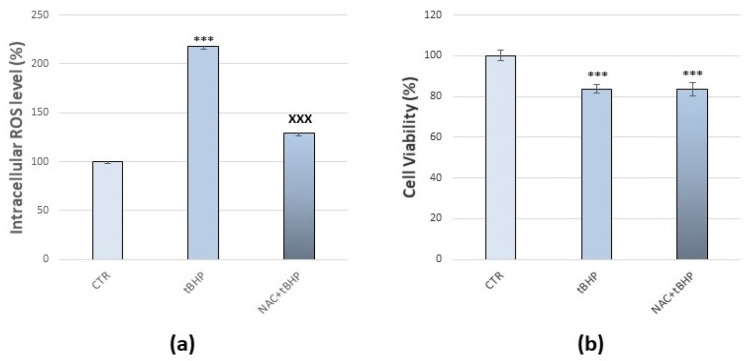
Effects of NAC against tBHP mediated oxidative stress in C6 glioma cells. (**a**) Intracellular DCF fluorescence intensity of cells treated with 50 µM tBHP for 30 min with and without pre-treatment with 5 mM NAC for 24 h. (**b**) Cell viability of cells treated with 50 µM tBHP for 30 min with and without pre-treatment with 5 mM NAC for 24 h. Values represent mean ± SE obtained from three independent experiments. *** *p* < 0.001 versus control, ^XXX^ *p* < 0.001 versus tBHP.

The assessment of ROS production and of cell viability are significant indicators of finding the degree of cytotoxicity, caused by any xenobiotics. First, we carried out tests to set up the appropriate method for the evaluation of ROS in our cellular model by tBHP, a known pro-oxidant [60]. Analysis by using DCFDA as a probe showed that treatment with 50 µM tBHP for 30 min caused the maximum ROS production, compared to the control (+118%, *p* < 0.001) (Figure 2a). After the assessment of cell viability by MTT assay, tBHP treatment resulted in a significant decrease in cell viability compared to the control (−16%, *p* < 0.001) (Figure 2b). However, the cells still had active cellular metabolism and produced ROS. After that, we evaluated the antioxidant effect of NAC in our cellular model [61]. C6 glioma cells were treated with NAC 5 mM [52], and analysis by using DCFDA as a probe showed that NAC treatment for 24 h resulted in a significant decrease (−20%, *p* < 0.001) in basal level of ROS without cytotoxic effects on cells, as demonstrated after the assessment of cellular viability by MTT assay (Figure S1).

Pre-treatment with NAC 5 mM for 24 h prevented tBHP-induced intracellular ROS production by keeping them at a much lower value (Figure 2a), while the antioxidant did not prevent cytotoxicity since cell viability remained around 80% (*p* < 0.001) (Figure 2b). Therefore, as our method of analysis was considered reliable for evaluating oxidative stress and assessing the antioxidant effects, we analysed the coffee phenolic metabolites antioxidant potential against DEP-induced oxidative stress compared to a known antioxidant, NAC.

### 3.2. Effects of Diesel Exhaust Particles (DEPs)

**Figure 3:**
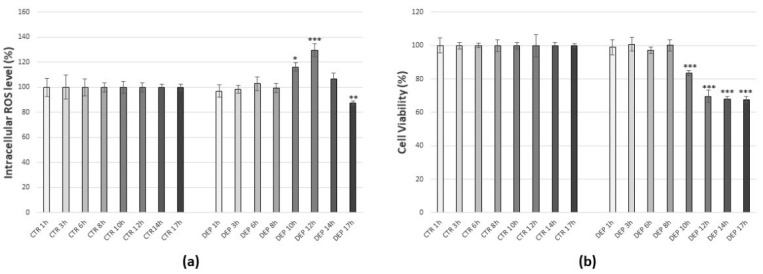
Time dependent effects of DEPs in C6 glioma cells. (**a**) Intracellular DCF fluorescence intensity of cells treated with 25 µg/mL DEPs for increasing times. (**b**) Cell viability of cells treated with 25 µg/mL DEPs for increasing times. Values represent Mean ± SE obtained from three independent experiments. * *p* < 0.05 versus control, ** *p* < 0.01 versus control, *** *p* < 0.001 versus control.

To assess the impact of atmospheric particulate matter on cells, DEPs were chosen as a surrogate model of the air pollution generated by motor vehicle traffic. C6 glioma cells were treated with 25 µg/mL DEPs [15,54] for increasing times (1 h, 3 h, 6 h, 8 h, 10 h, 12 h, 14 h, 17 h). Following analysis by DCFDA as a probe, a 25 µg/mL DEP treatment for 12 h showed a peak production of ROS, with an increase of 30 %29,6% compared to control (*p* < 0.001), although already after 10 h there was a rise in the ROS production (Figure 3a). Moreover, assessment of cell viability by MTT assay was carried out to verify the time-dependent effect of 25 µg/mL DEP treatment: after 10 h, cell viability began to decrease, and after 12 h it was 30% lower than the control (*p* < 0.001). However, the cells were still able to produce ROS, and the effects of DEPs on cell viability beyond this time (14 and 17 h) remained practically unaltered (Figure 3b). Consequently, for all the subsequent experiments, a dose of 25 µg/mL DEPs for 12 h was taken as the optimum to generate oxidative stress.

### 3.3. Effects of Coffee Phenolic Metabolites

**Figure 4:**
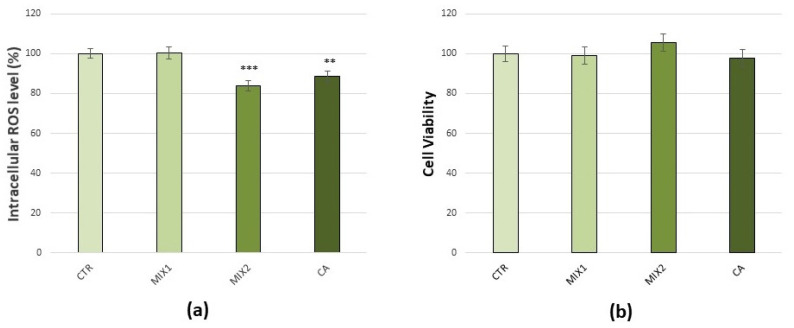
Effects of coffee phenolic metabolites in C6 glioma cells. (**a**) Intracellular DCF fluorescence intensity of cells treated with 0.5 µM Mix 1, 1 µM Mix 2, or 1 µM caffeic acid (CA) for 48 h. (**b**) Cell viability of cells treated with 0.5 µM Mix 1, 1 µM Mix 2, or 1 µM caffeic acid for 48 h. Values represent mean ± SE obtained from three independent experiments. ** *p* < 0.01 versus control, *** *p* < 0.001 versus control.

To verify that treatments with phenolic mixes were not harmful to the cells, evaluations of intracellular ROS production by DCFDA staining method (Figure 4a) and of cell viability by MTT assay (Figure 4b), were carried out. Results obtained indicated a decrease in basal production of ROS, after 1 µM Mix 2 or 1 μM caffeic acid (16%, *p* < 0.001 and 11.5%, *p* < 0.01, respectively), with no effect on cell viability. Consequently, the doses of 0.5 µM Mix 1, 1 µM Mix 2, and 1 μM caffeic acid for 48 h were considered as optimum and were used for all the subsequent experiments aimed to test their ability to prevent oxidative stress induced by DEPs.

### 3.4. Effects of Coffee Phenolic Metabolites against DEP-Mediated Oxidative Stress

**Figure 5:**
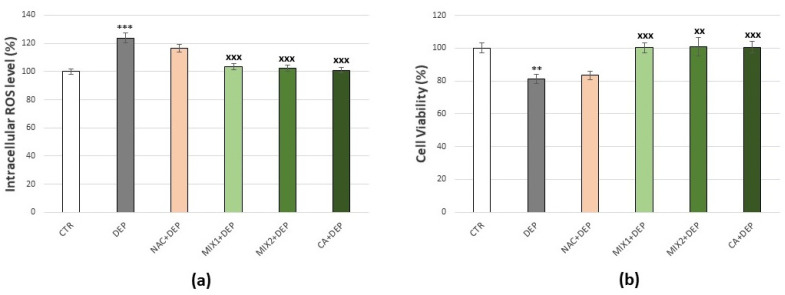
Effects of NAC and coffee phenolic metabolites against DEP-mediated oxidative stress in C6 glioma cells. (**a**) Intracellular DCF fluorescence intensity of cells treated with 25 µg/mL DEPs for 12 h with and without pre-treatment with 5 mM NAC for 24 h or 0.5 µM Mix 1, 1 µM Mix 2, and 1 µM caffeic acid (CA) for 48 h. (**b**) Cell viability of cells treated with 25 µg/mL DEPs for 12 h with and without pre-treatment with 5 mM NAC for 24 h or 0.5 µM Mix 1, 1 µM Mix 2, and 1 µM caffeic acid for 48 h. Values represent mean ± SE obtained from three independent experiments. ** *p* < 0.01 versus control, *** *p* < 0.001 versus control, ^XX^ *p* < 0.01 versus DEP, ^XXX^ *p* < 0.001 versus DEPs.

Results of the coffee phenolic metabolites’ effect against DEP-mediated oxidative stress are summarized in Figure 5. DEP exposure of cells at a dose of 25 µg/mL for 12 h caused ROS production and reduction in cell viability. In order to determine whether these effects could be prevented by coffee metabolites treatment, assessments of intracellular ROS production by DCFDA staining method (Figure 5a) and of cell viability by MTT assay (Figure 5b) were carried out. Actually, the incubation of C6 glioma cells with either 0.5 µM Mix 1, 1 µM Mix 2, or 1 μM caffeic acid for 48 h prior to DEP exposure prevented intracellular ROS production and cytotoxicity, preserving values similar to the control in both cases. The results showed that the effects caused by DEP exposure could not be prevented by the pre-treatment of the cells with NAC (Figure 5a,b).

### 3.5. Analysis of Oxidative Stress-Related Proteins

Some proteins that are known to be key regulators of cellular response to oxidative stress were analysed. To evaluate the different antioxidant strategies of NAC and coffee phenolic metabolites, each protein was evaluated following cell treatment with antioxidants (NAC, Mix 1, Mix 2, or caffeic acid) or with pro-oxidant (DEPs), and finally with the different antioxidants and subsequent exposure to DEPs.

**Figure 6:**
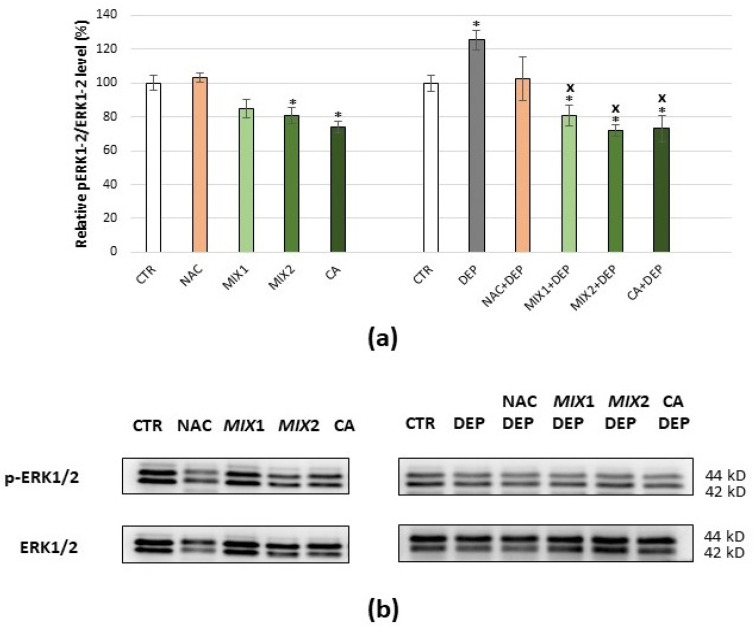
Immunoblotting analysis of total ERK1/2 phosphorylated/ERK1/2 ratio. (**a**) Protein was evaluated following cell treatment carried out only with antioxidants (5 mM NAC for 24 h, 0.5 µM Mix 1 or 1 µM Mix 2 or 1 µM caffeic acid (CA) for 48 h), only with pro-oxidant (25 µg/mL DEPs for 12 h), and finally with the different antioxidants and subsequent exposure to DEPs. Protein ratio is expressed as a percentage of the control. Values represent mean ± SE obtained from three independent experiments. * *p* < 0.05 versus control, ^X^ *p* < 0.05 versus DEP treatment. (**b**) Corresponding representative immunoblotting images.

ERK1/2 expression level and activation were evaluated by analysing the total ERK1/2 phosphorylated/ERK1/2 ratio, so that the ERK1/2 activation could be assessed following different treatments. NAC treatment did not determine differences in ERK1/2 activation when compared to the basal level, while coffee phenolic metabolites resulted in a modest decrease in ERK1/2 activation when compared to the basal level, which became significant with Mix 2 and caffeic acid (*p* < 0.05). Moreover, DEP exposure induced a significant increase in ERK1/2 activation (+25.5%, *p* < 0.05) compared to the control; this could be prevented by pre-treatment with all assessed antioxidants and in particular with coffee metabolites, which caused a further lowering compared to the control (Figure 6a,b).

Analysing the two isoforms ERK1/2 separately, the results obtained showed that ERK1/2 expression levels did not change significantly following any treatment, although pre-treatment with coffee phenolic metabolites appears to increase ERK2. Furthermore, the analysis of the p-ERK1/ERK1 and p-ERK2/ERK2 ratios showed that the pre-treatment with coffee phenolic metabolites preferentially inhibited ERK2 phosphorylation. Once again, NAC acted differently since pre-treatment with NAC preferentially inhibited ERK1 (see Figure S3 in Supplementary Materials).

**Figure 7:**
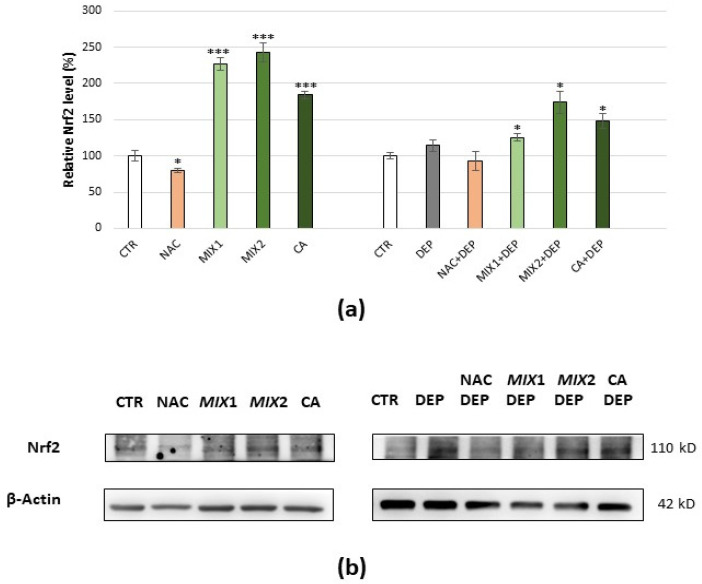
Immunoblotting analysis of Nrf2. (**a**) Protein was evaluated following cell treatment carried out only with antioxidants (5 mM NAC for 24 h, 0.5 µM Mix 1 or 1 µM Mix 2 or 1 µM caffeic acid (CA) for 48 h), only with pro-oxidant (25 µg/mL DEPs for 12 h), and finally with the different antioxidants and subsequent exposure to DEPs. Protein was normalized for the corresponding β-actin signal in each lane, and expressed as a percentage of the control. Values represent mean ± SE obtained from three independent experiments. * *p* < 0.05 versus control, *** *p* < 0.001 versus control. (**b**) Corresponding representative immunoblotting images.

Nrf2 expression level decreased following treatment with NAC (−21%, *p* < 0.05), while it increased following treatment with phenolic metabolites, i.e., +126%, +143%, and +84% (*p* < 0.001) after Mix 1, Mix 2, and caffeic acid exposure, respectively. DEP treatment also caused an increase in the Nrf2 level when compared to the control, although it was not significant after 12 h but after 10 h (+170%, *p* < 0.05) of exposure to DEPs (Figure S2). Moreover, pre-treatment with NAC counteracted the increase in Nrf2 level caused by DEPs, keeping it at around the control’s value, while pre-treatment with coffee metabolites caused a significant further increase in the protein, compared to the control (Figure 7a,b).

**Figure 8:**
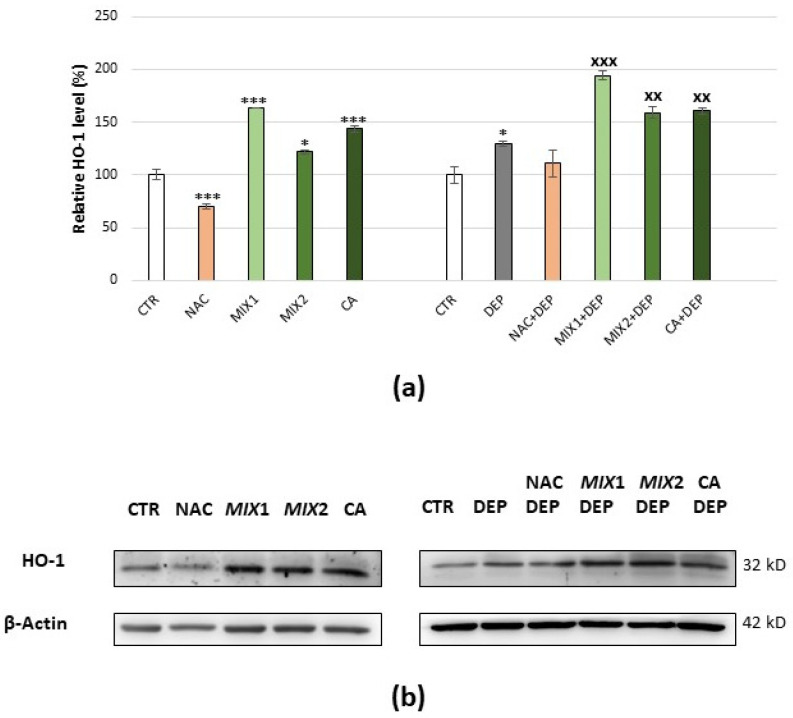
Immunoblotting analysis of HO-1. (**a**) Protein was evaluated following cell treatment carried out only with antioxidants (5 mM NAC for 24 h, 0.5 µM Mix 1 or 1 µM Mix 2 or 1 µM caffeic acid (CA) for 48 h), only with pro-oxidant (25 µg/mL DEPs for 12 h), and finally with the different antioxidants and subsequent exposure to DEPs. Protein was normalized for the corresponding β-actin signal in each lane, and expressed as a percentage of the control. Values represent mean ± SE obtained from three independent experiments. * *p* < 0.05 versus control, *** *p* < 0.001 versus control, ^XX^ *p* < 0.01 versus DEPs, ^XXX^ *p* < 0.001 versus DEPs. (**b**) Corresponding representative immunoblotting images.

The trend of HO-1 was very similar to that of Nrf2. NAC treatment showed a significant decrease in HO-1 comparing to the basal level (−30%, *p* < 0.001), while following coffee metabolite treatment, HO-1 was significantly higher than the basal level. Cells incubated with DEPs displayed a significant increase in HO-1 (+33%, *p* < 0.05) compared to the control, and a very different trend was observed with the different antioxidant pre-treatments. Indeed, pre-treatment with NAC kept HO-1 at around the control value, while pre-treatment with phenolic metabolites resulted in a further increase in the protein (Figure 8a,b).

**Figure 9:**
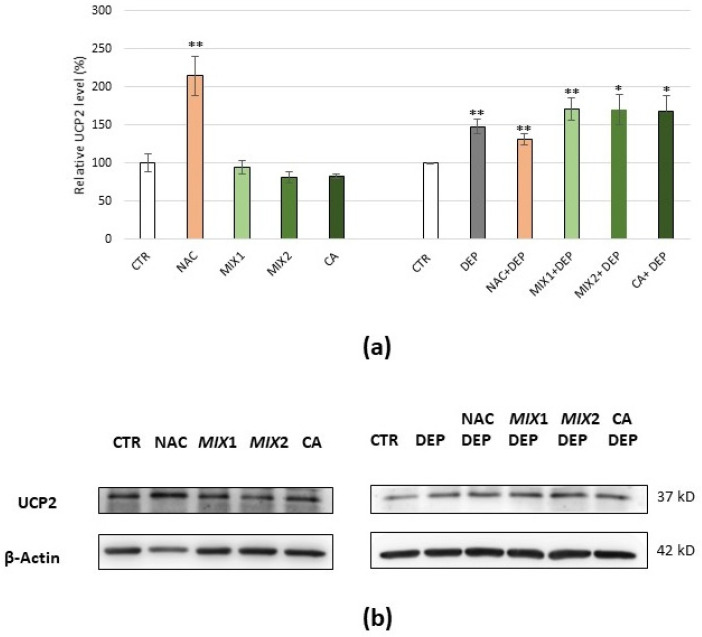
Immunoblotting analysis of UCP2. (**a**) Protein was evaluated following cell treatment carried out only with antioxidants (5 mM NAC for 24 h, 0.5 µM Mix 1 or 1 µM Mix 2 or 1 µM caffeic acid (CA) for 48 h), only with pro-oxidant (25 µg/mL DEPs for 12 h), and finally with the different antioxidants and subsequent exposure to DEPs. Protein was normalized for the corresponding β-actin signal in each lane and expressed as a percentage of the control. Values represent mean ± SE obtained from three independent experiments. * *p* < 0.05 versus control, ** *p* < 0.01 versus control. (**b**) Corresponding representative immunoblotting images.

Finally, the UCP2 expression level was analysed. Cells treatment with NAC caused a significant increase in the UCP2 basal level compared to the control (*p* < 0.01), while treatment with coffee phenolic metabolites resulted in a slight decrease in the protein. Moreover, UCP2 increased significantly compared to the control following DEP exposure (+48%, *p* < 0.01). Interestingly, the increase in UCP2 was less evident in the case of pre-treatment with NAC (+31%, *p* < 0.01), while it became even more significant following pre-treatment with Mix 1, Mix 2, and caffeic acid (+70%, *p* < 0.01; +70 %, *p* < 0.05; +65.5%, *p* < 0.05, respectively) (Figure 9a,b).

## 4. Discussion

During the last few decades, interest in redox signalling studies has increased because of the involvement of ROS in deleterious effects on macromolecules. As ROS are harmful to cellular structure and activity, cells respond by triggering antioxidant and cytoprotective mechanisms [62]. Recently, it was shown in China that a supplement in the form of an herbal product composed of ginseng, Lilii Bulbus, and poria has anti-inflammatory and antioxidant activity, and it offers protective effects on PM2.5-induced damage to cardiopulmonary health [63].

The contributory role of (poly) phenols to protection against oxidative stress and to the modulation of neurodegeneration is being extensively investigated [28,29,30]. Their mechanisms of in vivo action depend on the extent to which they are metabolized and conjugated during absorption [64,65].

Therefore, in the present study we used DEPs as a model of air pollution with pro-oxidant effects in C6 glioma cells [15], and we analysed the action of different phenolic metabolites at concentrations that are physiologically achievable upon coffee consumption in a putative antioxidant strategy, while comparing it to that of a known antioxidant, NAC.

### 4.1. Antioxidant Strategies of Coffee Phenolic Metabolites against DEP-Induced Oxidative Stress

It is known that concentrations of 5–50 µg/mL of DEPs can reach the CNS of individuals exposed to air pollution daily [53]. Taking this value as a guideline, the C6 glioma cells were exposed to 25 µg/mL DEPs [15] for increasing lengths of time to determine the optimal treatment that would generate oxidative stress.

Based on the results, the treatment with 25 µg/mL DEPs for 12 h was taken as the optimum for assessing the antioxidant effects of NAC and coffee metabolites against DEP-induced oxidative stress. The pre-treatment of cells with NAC followed by DEP exposure was not effective neither in contrasting the increase in ROS nor in preventing the DEP-mediated cytotoxicity. Therefore, NAC cannot have any effect on DEP-induced oxidative stress.

On the contrary, the pre-treatment of cells with coffee metabolites (0.5 µM Mix 1, 1 µM Mix 2, or 1 μM caffeic acid) for 48 h followed by DEP exposure successfully prevented oxidative stress and cytotoxicity induced by DEP treatment. Indeed, ROS production and cell viability were kept almost at the control’s value. Evidently, NAC and coffee phenolic metabolites adopt different mechanisms to exercise their antioxidant action. NAC, being a precursor of GSH [66] that reacts directly with ROS by decreasing their level, counteracts the oxidative stress induced by tBHP in this way, although it cannot hinder its cytotoxic effect. However, NAC pre-treatment is ineffective in reducing DEP-induced oxidative stress, likely due to its typical composition. Indeed, DEPs consist of a mixture of inorganic and organic compounds adsorbed on a carbonaceous nucleus [12]; this could contribute differently to oxidative stress induction acting at different levels.

It is noteworthy that coffee phenolic metabolites were effective in preventing the adverse effects caused by DEP exposure. This result is promising, as these circulating metabolites of coffee hydroxycinnamates counteracted the production of ROS induced by DEPs and its cytotoxicity at very low concentrations.

### 4.2. Proteins Involvement in Defense against Oxidative Stress

Literature data showed that the MEK-ERK1/2 pathway is involved in the antioxidant response in C6 glioma cells after DEP exposure [15]. In particular, ROS contribute together with DEPs themselves to the induction of the MAPKs pathway, involving MEK and ERK, resulting in Nrf2 activation, thus causing an increase in antioxidant enzymes such as HO-1 [19,20].

Moreover, it is known that subcellular DEP targets include mitochondria that are both the major intracellular source and the target of oxidative stress [22]. Mitochondrial uncoupling protein 2 (UCP2), a proton transporter located in the inner mitochondrial membrane, has the capability of ameliorating ROS generation by dissipating the mitochondrial proton gradient and mitochondrial membrane potential [67]. Therefore, we evaluated proteins mediating the downregulation of ROS following DEP exposure, which include ERK1/2, Nrf2, HO-1, and UCP2.

The results obtained by protein analysis reinforced our hypothesis that NAC and coffee phenolic metabolites adopted different mechanisms to exercise their antioxidant action. Following cells’ exposure to DEPs, an increase in ERK1/2 activation was observed, and the pre-treatment with all the evaluated antioxidants (NAC, Mix 1, Mix 2, or caffeic acid) prevented it. In particular, coffee phenolic metabolites resulted in a modest but significant decrease in ERK1/2 activation.

Our results are in agreement with data in the literature showing that PM and/or DEPs induce the activation of ERK1/2 [68,69] even for a long time [70], and that resveratrol, a well-known phenolic compound, reduces the activation of ERK1/2 and the production of ROS [68]. Li and coauthors demonstrated that caffeic acid inhibits both JAK/STAT and ERK1/2 pathways, as well as cell proliferation [71]. Moreover, it was shown that nobiletin, a methylated flavonoid found in citrus peels, suppresses the proliferation of C6 rat glioma cells by inhibiting RAS activity and subsequently reducing MEK/ERK signalling [72]. The ERK1/2 expression levels did not change significantly following any treatment, while the p-ERK2/ERK2 ratio decreased after pre-treatment with coffee phenolic metabolites. However, despite the large number of studies assessing the functional differences between ERK1 and ERK2 so far, they are still under debate, and very little is known regarding the specific role and the in vivo targets of the two ERK isoforms [73]. Although Nrf2 activation may depend on regulation of both Keap1-dependent and the Keap1-independent pathways (i.e., Nrf2 phosphorylation by activated ERK1/2), the authors in [15,74] showed that the Keap1-independent pathway is essential in Nrf2 activation by DEPs. In agreement with these studies, an increase in the Nrf2 level following ERK1/2 activation by DEPs was registered.

Consistent with the results observed for Nrf2, the HO-1 level increased following cells’ exposure to DEPs, as the cell’s self-protective response to ROS. However, after exposure of the cells to various antioxidants, opposite effects were observed: while NAC treatment caused a decrease in Nrf2 level expression and in its downstream protein HO-1, coffee phenolic metabolites seemed to activate this pathway. We observed that after cells’ pre-treatment with NAC and successive DEP incubation, the Nrf2 level expression and its downstream protein HO-1 did not increase, suggesting that NAC could prevent DEPs from activating the Nrf2/HO-1 signal pathway. These results were in agreement with those obtained in a study on silver nanoparticle-mediated cytotoxicity, in which nano-Ag-treated cells showed an increase in the protein level of Nrf2, whereas this increase was blunted by NAC pre-treatment [75]. On the contrary, pre-treatment of cells with coffee metabolites induced an increase in Nrf2 and HO-1 level expression but independent of ERK1–2 pathway.

Since it is known that the phenolic induction of Nrf2 and its downstream enzymes may be Keap1-dependent or Keap1-independent [76], we cannot exclude the possibility that the observed induction of Nrf2 is dependent on ERK1/2 activation ending before the 48 h of coffee metabolites incubation. However, the regulatory mechanisms involved in Nrf2 activation are not yet fully understood. Indeed, it was shown that mangiferin, a polyphenol extracted from the mango plant, with antioxidant and cytoprotective activities, prolonged the half-life of the Nrf2 protein by inhibiting its ubiquitination and degradation, which led to Nrf2 protein accumulation in stressed cells. Therefore, it was suggested that Nrf2 activation is dependent on increasing Nrf2 protein stability [21,77,78,79]. These results suggested once again that the two antioxidant strategies are different.

Finally, we observed UCP2 induction following the oxidative insult by DEPs, which may suggest that in response to DEPs, proton conductance through UCP2 increased, providing a negative feedback loop to try to limit further mitochondrial ROS formation [80,81]. It is known that the cellular effect of DEPs is very complex, and mitochondria are highly sensitive to environmental toxicants. In particular, PM2.5 was shown to accumulate within mitochondria and further disrupt mitochondrial membrane potential, damaging mitochondrial structure and function. Several studies have also suggested that metals and PAHs, rich in DEP, exert their toxicity through different mechanisms involving mitochondria [82].

Again, NAC and coffee phenolic metabolites seemed to have different antioxidant strategies. Indeed, NAC caused a significant increase in the UCP2 basal level, and coffee metabolites induced a slight decrease in the UCP2 basal level. Dietary flavonoids were shown to be putative inducers of the transcription factors Nrf2, FoxO, and PPARγ [83]. It is also known that the PPAR-γ/PGC-1α expression improves mitochondrial decoupling, which reduces mitochondrial membrane potential and reactive oxygen species (ROS) production, oxidative damage, and mitochondrial calcium overload through the induction of UCP2 [84]. Our results indicated that UCP2 is physiologically important in modulating the generation of ROS from mitochondria of C6 glioma cells exposed to DEPs. Indeed, DEP administration caused an increase in the UCP2 level; one explanation is that it may have tried to implement an antioxidant strategy since it is known that UCP2 induction participates in the defence against oxidants [85]. However, the cells were unable to counteract ROS production by DEPs. Instead, after pre-treatment with coffee phenolic metabolites and subsequent exposure to DEP, the UCP2 level increased, countering ROS production by DEPs. In literature, it was already observed that over-expression of UCP2 inhibits ROS generation [27,86].

Pre-treatment with coffee metabolites was also able to counteract DEP-induced cytotoxicity, as demonstrated by the restoration of cell viability to the control value. This result is in agreement with data in the literature in which over-expression of the UCP2 was accompanied by increased cell survival after H_2_O_2_ exposure [86]. On the contrary, NAC caused a significant increase in the UCP2 basal level, which could have reduced quickly, since NAC appears to have an inhibitory effect on the increase in UCP2 caused by exposure to DEP. It is known that the UCP2 protein level increases and decreases in a very short time [87]. We can speculate that NAC prevented the induction of UCP2 by DEP, as already observed in the literature in mice after treatment with NAC and LPS [86], thus counteracting the inhibition of ROS generation.

Based on the data obtained, the increase in cellular components mediating the downregulation of ROS following cells’ exposure to DEPs, such as HO-1 and UCP2, can be interpreted as a defence mechanism against DEP-induced oxidative stress. This pathway appears to be counteracted by pre-treatment with NAC, while it seems to be supported by pre-treatment with coffee phenolic metabolites, which consequently provide an effective defence against DEP-induced oxidative stress.

However, it is known that exposure to air pollution particles is associated with ROS production, inflammation, and oxidative DNA damage [11], and so we cannot exclude the possibility of anti-inflammatory action and protective action taking place on DNA by coffee phenolic metabolites. Moreover, as reported by Martini et al. (2016) [88], coffee consumption can improve protection against DNA damage, especially following regular/repeated intake, and Radhot et al. (2013) [89] showed that phenolic and caffeine metabolites are active in reducing the amount of radical-induced strand breaks, most likely by an antioxidant mechanism between such active compounds and DNA radicals.

## 5. Conclusions

In literature, C6 glioma cells after exposure to diesel exhaust particles were found to activate anti-oxidant pathways to contrast the oxidative status induced by DEP treatment [15,19,20] involving MEK and ERK1/2, and resulting in Nrf2 activation, thus causing an increase in antioxidant enzymes such as HO-1 and UCP2.

However, the data obtained in our study showed that the cells failed to be effective in counteracting the oxidative stress that is DEP induced. The novelty of this work is that after analysing the point of maximum production of DEP-induced ROS in our cellular model, we studied the potential antioxidant effect of the coffee phenolic metabolites (not coffee phenolic compounds) by evaluating their effect on proteins known as antioxidant markers causally linked to ROS production by DEPs.

In conclusion, this study showed that the phenolic metabolites of coffee provide an effective defence against DEP-induced oxidative stress by supporting the antioxidant strategy initiated by cells following exposure to diesel exhaust particles, thus making them able to counteract the damage. In this perspective, coffee phenolic metabolites can be promising molecules to protect against oxidative stress induced by daily exposure to air pollution generated by motor vehicle traffic.

## Data Availability

The datasets used and/or analysed during the current study available from the corresponding author on reasonable request.

## Supplementary Materials

The following are available online at https://www.mdpi.com/article/10.3390/antiox10081169/s1, Figure S1: Effects of NAC in C6 glioma cells. (a) Intracellular DCF fluorescence intensity of cells treated with 5 mM NAC for 2 or 24h. (b) Cell viability of cells treated with 5 mM NAC for 2 or 24h, Figure S2: Representative immunoblotting of (a) Nrf2, evaluated following cells treatment carried out with 25 μg/mL DEP for increasing times and of (b) corresponding actin, Figure S3: (a) Analysis of total ERK1-2/Actin. (b) Analysis of ERK1-2 phosphorylated/ERK1-2 ratio.
