# Supplementary material for: Study of the Antioxidant Effects of Coffee Phenolic Metabolites on C6 Glioma Cells Exposed to Diesel Exhaust Particles
Source: Antioxidants (Basel). 2021 Jul 23;10(8):1169. doi: 10.3390/antiox10081169 (PMC8388867; doi:10.3390/antiox10081169)
Supplement: Supplementary file 1 [file antioxidants-10-01169-s001.zip › antioxidants-1280889-SI.pdf]

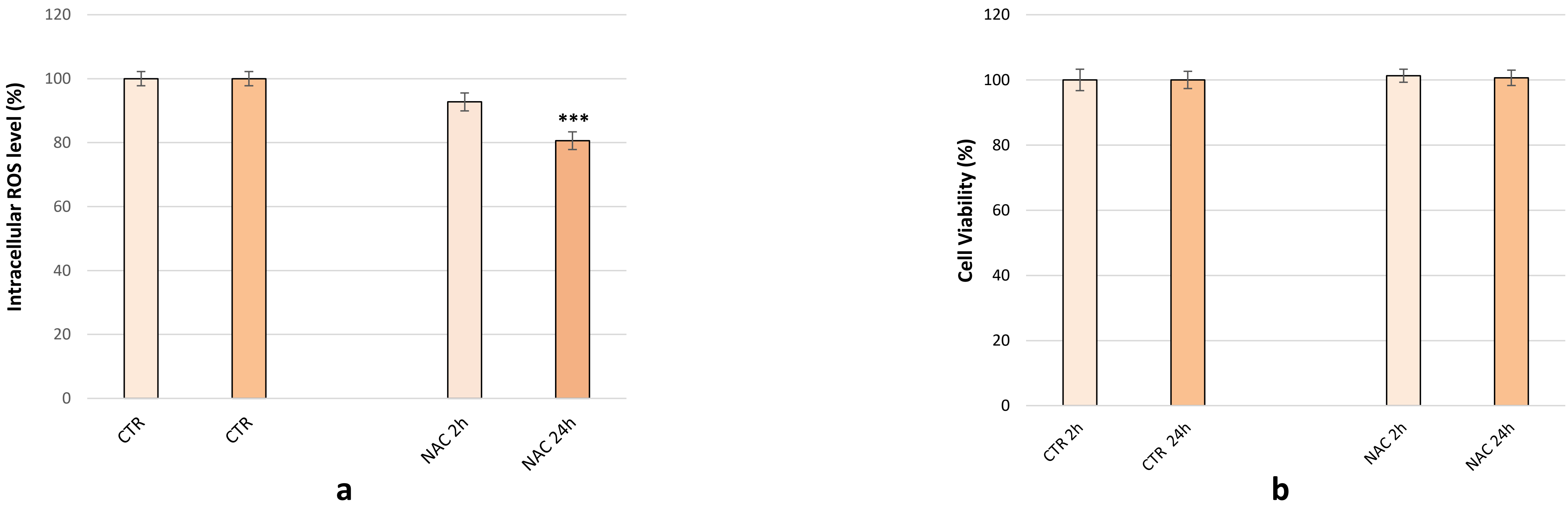

Figure S1. Effects of NAC in C6 glioma cells. (a) Intracellular DCF fluorescence intensity of cells treated with 5 mM NAC for 2 or 24h. (b) Cell viability of cells treated with 5 mM NAC for 2 or 24h. $p < 0,001$ vs CTR

Nrf2

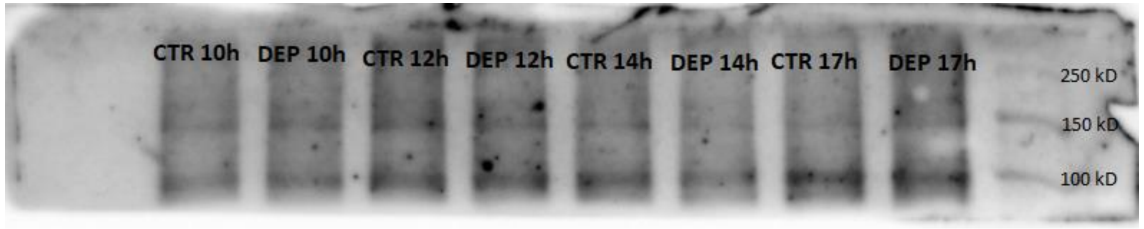

a

Actin

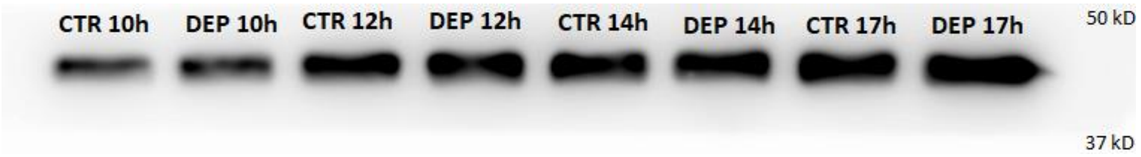

b

Figure S2. Representative immunoblotting of (a) Nrf2, evaluated following cells treatment carried out with 25 µg/ml DEP for increasing times and of (b) corresponding actin.

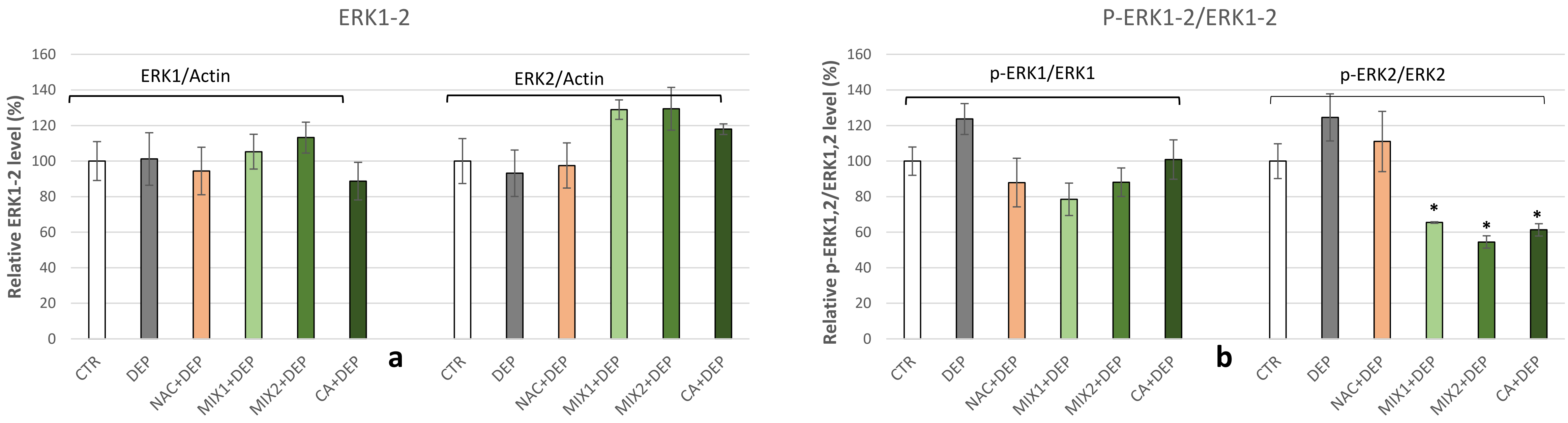

Figure S3. a) Analysis of total ERK1-2/Actin. b) Analysis of ERK1-2 phosphorelated/ERK1-2 ratio.
Protein ratio has been expressed as a percentage of the control.
Values represent Mean ± SE obtained from three independent experiments.
*$p < 0.05$ versus control
